# From inputs to outputs: an analysis of the changes to learning outcomes for dental undergraduate education in the UK

**DOI:** 10.1038/s41415-022-3873-y

**Published:** 2022-01-28

**Authors:** Helen Mather, Heidi Bateman, John Taylor, Christopher Vernazza, Charlotte Rothwell, Giles McCracken, Janice Ellis

**Affiliations:** 4141594585001 School of Dental Sciences, Faculty of Medical Sciences, Newcastle University, Newcastle Upon Tyne, UK; 4141594585002 Population Health Sciences Institute, Newcastle University, Newcastle Upon Tyne, UK

## Abstract

**Introduction/aims ***Preparing for practice *(PfP) was thought to represent a significant shift in the expectations of dental undergraduates compared to its predecessor, *The first five years* (TFFY). This project aimed to explore requirement changes by comparing learning outcomes for undergraduate dentists in these two documents. Changes in curriculum requirements defining clinical, professional, or a blend of these skills were also investigated.

**Methods **Curriculum mapping was used to compare learning outcomes in PfP to requirements in TFFY.

**Results **The total number of learning outcomes increased from 101 to 149 in PfP compared to TFFY. There was a proportional reduction in outcomes describing clinical skills and an increase in the proportion of outcomes describing professional and blended skills. Three TFFY requirements did not appear in PfP and a further 23 learning outcomes in PfP were absent in TFFY.

**Conclusions **In the transition from TFFY to PfP, there has been an overall increase in the number of outcomes graduates must attain before they can register with the General Dental Council. There are more outcomes defining professionalism which subsequently has resulted in proportional but not actual decrease in outcomes related to clinical skills. While there is uncertainty over how schools have managed curricula to incorporate these changes and thus whether the perception of graduate preparedness can be directly attributable to these changes, it is timely to consider any changes within dental learning outcomes in the context of preparedness concerns.

## Key points

- Highlights developments in content and structure of learning outcomes for dentists in the revision from *The first five years* to *Preparing for practice.*
- Considers how professional and clinical skills have been incorporated into curricula.
- Acknowledges the importance of stakeholder involvement when revising learning outcomes so that there is a clear understanding of the skillset of the dental graduate.

## Background

Curricula for professional courses are commonly described by regulators of professions, such as the General Dental Council (GDC) or the General Medical Council (GMC) in the UK, and in recent years the accepted pedagogy underpinning such curricula has tended to move to an outcomes-based model. Learning outcomes will often align to professional standards that the student must demonstrate by graduation to enable full or partial registration with their regulator, thus enabling them to practise within their chosen profession. Regulator-defined learning outcomes are subsequently used by higher education providers to inform curricula content, design and assessment. Regardless of the pedagogical approach chosen, multiple stakeholders may have a view on what skills and attributes the new graduate should have and how these should be defined, which will in turn drive the curricula described.

As the registrant body for UK dentists, the GDC accredits and quality-assures undergraduate dental education programmes. The GDC describes the expected outcomes of undergraduate programmes in *Preparing for practice: dental team learning outcomes for registration* (PfP), where learning outcomes are specified for each registrant group.^1^^,^^2^ PfP replaces the GDC's previous policy document *The first five years* (TFFY), which provided recommendations on curriculum content in addition to the learning outcomes for dentists.^3^^,^^4^ Higher education institutions must ensure their undergraduate programmes demonstrate satisfactory attainment of the GDC learning outcome for graduates to be permitted onto the UK dentists' register.

On completion of a GDC-accredited undergraduate programme, UK dental graduates can apply for full registration with the GDC. The majority of UK dental graduates will complete dental foundation training (99.6% in 2019),^5^ which is strongly endorsed and mandated for those who wish to practise within the NHS; however, a minority will progress directly into private practice, move abroad, complete further qualifications or pursue a career outside of the NHS or dentistry itself. Irrespective of the route taken, graduates are arguably entering into a profession which continues to undergo considerable challenge and development; this has been further exacerbated by the ongoing COVID-19 pandemic.

Within the profession, concerns have been expressed as to the standard of new graduates and the extent to which they are prepared for clinical practice.^6^^,^^7^^,^^8^^,^^9^^,^^10^^,^^11^^,^^12^^,^^13^^,^^14^^,^^15^ Such concerns are certainly not new,^16^ but publications regarding this topic have increased in the last 10-15 years which includes the period during which PfP was introduced in 2012.^2^ New graduates are regularly compared to their predecessors,^17^ and studies involving foundation dentists and educational supervisors (previously vocational dental practitioners and trainers) have suggested that graduates lack clinical experience, specifically in more complex procedures such as molar endodontics and fixed prosthodontics.^9^^,^^10^^,^^12^^,^^14^^,^^15^^,^^18^^,^^19^^,^^20^^,^^21^ It has also been suggested that a greater curricular emphasis on 'soft skills', such as health promotion, professionalism and communication, may be at the detriment of clinical skill development in undergraduates.^14^ It is hypothesised that if such changes have occurred, they are likely to have been influenced by educational providers' interpretation of the learning outcomes described in PfP, and the subsequent implementation of curriculum review and development. However, the nature of the changes that occurred in the revision of TFFY and the formulation of PfP have not been explored to date.

## Aims

To compare the expected outcomes for dental graduates as described by the learning outcomes in PfP^2^ with those educational requirements in the second edition of TFFY^4^ and to consider changes in the outcomes which defined clinical skills, professional skills or a blend of clinical and professional skills.

## Methods

This was a mapping study cross-referencing and comparing the learning outcomes in PfP to those in TFFY.

### Data sources

Educational requirements from the second edition of TFFY^4^ and learning outcomes from the first publication of PfP^2^ for dentists were identified. Educational requirements from TFFY, which are described as learning outcomes in the document, are preceded by the prefixes 'be competent in', 'have knowledge of' or 'be familiar with'. These were included in the mapping and were numbered in the order presented in the 'Dental domains' section of TFFY.^4^ In PfP, learning outcomes for dentists were identified under the following domains: 'clinical', 'communication', 'professionalism' and 'management and leadership'.^2^ The organisation of the learning outcomes by domain and sub-domain in TFFY and PfP is shown in Tables 1 and 2. Both sets of learning outcomes were inputted into an Excel spreadsheet with each requirement from TFFY corresponding to a column heading, and each learning outcome from PfP corresponding to a row heading.

**Table 1:** Organisation of educational requirements in the 'Dental domains' section of TFFY; educational requirements for mapping were identified in the order they appeared in these domains. Adapted from requirements listed in the 'Dental domains'^4^

*The first five years* 'Dental domains'	Number of educational requirements
Clinical skills	13
Practical procedures	22
Patient investigation	5
Patient management	20
Health promotion and disease prevention	5
Communication	4
Data and information handling skills	3
Understanding of basic and clinical sciences and underlying principles	10
Appropriate attitudes, ethical understanding and legal responsibilities	3
Appropriate decision-making, clinical reasoning and judgement	8
Professional development	4
Personal development	4

**Table 2:** Organisation of learning outcomes for dentists by domain and sub-domain, adapted from the list of learning outcomes for dentists in PfP^2^

Domain	Sub-domain	Number of learning outcomes
Clinical	Individual patient care: foundations of practice	13
	Individual patient care: comprehensive patient assessment	6
	Individual patient care: diagnosis	2
	Individual patient care: treatment planning	6
	Individual patient care: patient management	11
	Individual patient care: patient and public safety	8
	Individual patient care: treatment of acute oral conditions	4
	Individual patient care: health promotion and disease prevention	7
	Individual patient care: management of periodontal disease	6
	Individual patient care: hard and soft tissue disease	8
	Individual patient care: management of the developing and developed dentition	7
	Individual patient care: restoration and replacement of teeth	12
	Population-based health and care	5
Communication	Patients, their representatives and the public	4
	Team and the wider healthcare environment	4
	Generic communication skills	5
Professionalism	Patients and the public	5
	Ethical and legal	4
	Teamwork	3
	Development of self and others	7
Management and leadership	Managing self	8
	Managing and working with others	8
	Managing the clinical and working environment	6

### Initial curriculum mapping

**Fig. 1:**
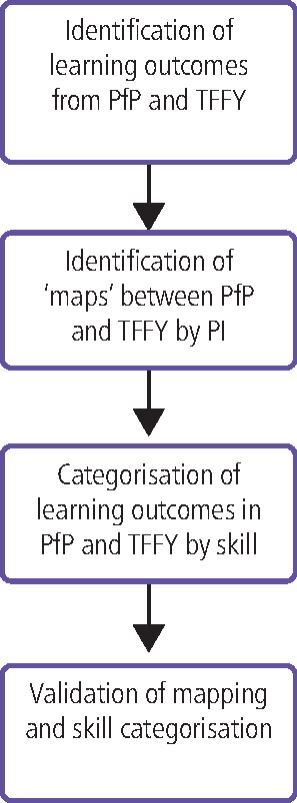
Flowchart of the curriculum mapping process

An overview of the curriculum mapping process is shown in Figure 1. Each educational requirement in TFFY was sequentially assessed by the principal investigator (HM) to identify learning outcomes in PfP which encompassed the same skill, knowledge or attitude with outcomes considered to fully, partially, slightly, or not at all map (Table 3). It was accepted PfP learning outcomes could map to more than one TFFY outcome. Where requirements or learning outcomes in either TFFY or PfP had no apparent cross mapping, further scrutiny using the reverse direction of mapping was undertaken.

**Table 3:** Descriptors used in the mapping of learning outcomes from PfP (on) to educational requirements in TFFY

Descriptor	Explanation
Fully maps	The skill, knowledge, or attribute in TFFY requirement is fully covered by PfP learning outcome(s)
Partially maps	The skill, knowledge or attribute described in TFFY is partially covered by PfP learning outcome(s), but the link is not sufficient to fully map
Slightly maps	The skill, knowledge or attitude described in TFFY can only be considered to be minimally covered by the PfP learning outcome(s); however, the mapping is still considered to be of significance
Does not map	There are no learning outcomes in PfP that map to an outcome in TFFY

### Categorising requirements and learning outcomes

Educational requirements (TFFY) and learning outcomes (PfP) were categorised into skill type by the principal investigator (HM) using the definitions shown in Table 4. These were developed using available definitions from TFFY and PfP.^2^^,^^4^

**Table 4:** Definitions of clinical, professional and blended skill categories, adapted from available definitions in TFFY and PfP with examples from TFFY and PfP^2^^,^^4^

Type of learning outcome or requirement	Definition	Example of educational requirement from *The first five years*	Example of learning outcome from *Preparing for practice*
Clinical	The skills (and supporting knowledge/attributes) necessary to provide safe patient care	Be competent at obtaining a detailed history of the patient's dental state	1.2.1 Obtain, record and interpret a comprehensive and contemporaneous patient history
Professional	The skills (and supporting knowledge/attributes) required to practise ethically and act in the best interests of the patient	Be familiar with the legal and ethical obligations of registered dental practitioners	1.7.1 Treat all patients with equality, respect and dignity
Blended	A combination of the skills (and supporting knowledge/attributes) that constitute both clinical and professional skills	Have knowledge of managing patients from different social and ethnic backgrounds	1.8.3 Recognise and take responsibility for the quality of services and devices provided to the patient

### Validation and revision of mapping and skill categorisation

Following initial mapping and categorisation, a validation process took place with two other members of academic staff from the School of Dental Sciences. Considering the reflexivity of the validation team, each member had different experiences and perspectives that they brought to the process; this ensured non-clinical and clinical components of educational requirements and learning outcomes were considered equally. Each validation team member had experience of designing and implementing dental curricula and use of learning outcomes. One team member had experience of delivering outcomes in TFFY and PfP, exclusively in the non-clinical training of undergraduates. The second validation team member, in addition to clinical training of dentists, has expertise and experience in curriculum mapping and blueprinting. The principal investigator currently delivers teaching to align to learning outcomes in the revised version of PfP and was an undergraduate at the time of the transition from the interim edition of TFFY to PfP. Taking the same approach to mapping and categorisation, the team validated the entirety of the mapping and a sample of the categorisation of requirements and learning outcomes. Reviewing the results after the validation process allowed for recursive analysis.

## Results

*Preparing for practice* (2012) contains 149 learning outcomes which are divided into four domains (clinical, communication, professionalism, and management and leadership) with 23 sub-domains. The second edition of *The first five years* has 101 education al requirements divided into 12 domains.

### Categorisation of learning outcomes and requirements by skill

There were clear differences in the proportional representation for the different categories of learning outcomes when the two policy documents were compared, as shown in Table 5.

**Table 5:** Proportions of skill type in TFFY and PfP

Type of learning outcome or requirement	TFFY	PfP
	N (out of 101) (%)	N (out of 149) (%)
Clinical	84 (83%)	88 (59%)
Professional	10 (10%)	37 (25%)
Blended	7 (7%)	24 (16%)

### Mapping of learning outcomes to educational requirements

There were three outcomes in TFFY to which no outcomes from PfP could be mapped (Table 6).

**Table 6:** Educational requirements from TFFY not mapped to by learning outcomes in PfP

Domain	Learning outcome	Skill type
Practical procedures	Have the knowledge to design, insert and adjust space maintainers	Clinical
Practical procedures	Have the knowledge to design, insert and adjust active removable appliances to move a single tooth or correct a cross bite	Clinical
Patient management	Have knowledge of dental problems that may manifest themselves in older patients and of the principles involving the management of such problems	Clinical

There were 23 learning outcomes in PfP that did not map to any outcomes from TFFY (Table 7); 12 of these were defined as clinical skills, 5 were professional skills and 6 were blended skills. Sixteen of the outcomes were within the 'clinical skills' domain, four within the 'management and leadership' domain, two in the 'communication' domain and one in the 'professionalism' domain.

**Table 7:** PfP learning outcomes that did not map to educational requirements in TFFY

Domain	Preparing for practice learning outcome	Skill type
Clinical	1.1.12 Explain the principles of epidemiology and critically evaluate their application to patient management	Clinical
	1.2.6 Discuss the importance of each component of the patient assessment process	Clinical
	1.5.2 Describe the range of orthodox complementary and alternative therapies that may impact on patient management	Clinical
	1.5.6 Critically evaluate the treatment planning process	Clinical
	1.7.3 Monitor and review treatment outcomes	Clinical
	1.7.9 Explain the role and organisation of referral networks, clinical guidelines and policies and local variation	Blended
	1.7.11 Critically evaluate all components of patient management	Clinical
	1.8.3 Recognise and take responsibility for the quality of services and devices provided to the patient	Blended
	1.8.4 Explain the responsibilities and limitations of delegating to other members of the dental team	Blended
	1.8.8 Identify the signs of abuse or neglect, explain local and national systems that safeguard welfare and understand how to raise concerns and act accordingly	Blended
	1.9.1 Recognise and manage patients' acute orofacial and dental pain	Clinical
	1.9.4 Identify the need for and make arrangements for follow-up care	Clinical
	1.11.4 Monitor and record changes in periodontal health on a regular basis using appropriate methods	Clinical
	1.13.3 Identify and explain developmental or acquired occlusal abnormalities	Clinical
	1.14.3 Create an oral environment where restoration or replacement of the tooth is viable	Clinical
	1.14.7 Recognise the role of surgical management of periradicular disease	Clinical
Communication	4.2 Explain the role of appraisal, training and review of colleagues, giving and receiving effective feedback	Professional
	4.3 Give and receive feedback effectively to other members of the team	Professional
Professionalism	9.6 Accurately assess their own capabilities and limitations in the interest of high-quality patient care and seek advice from supervisors or colleagues where appropriate	Professional
Management and leadership	10.2 Effectively manage their own time and resources	Professional
	10.4 Recognise the significance of their own management and leadership role and the range of skills and knowledge required to do this safely	Professional
	11.4 Where appropriate, lead, manage and take professional responsibility for the actions of colleagues and other members of the team involved in patient care	Blended
	12.6 Describe the implications of the wider heath economy and external influences	Blended

For 25 of the educational requirements in TFFY, one learning outcome from PfP mapped to one requirement from TFFY. An example of this is: TFFY requirement 'be competent at obtaining a detailed history of the patient's dental state' was mapped to by PfP learning outcome 1.2.2: 'Recognise the importance of and record a comprehensive and contemporaneous patient history'.

However, for the remaining 76 TFFY requirements, there was not a one-to-one relationship with PfP learning outcomes, and in some cases, multiple PfP learning outcomes could be mapped to each TFFY requirement. As an example, the TFFY requirement 'be familiar with the legal and ethical obligations of registered dental practitioners' was mapped to by 33 learning outcomes from PfP.

In total, there were 298 instances where PfP learning outcomes mapped to TFFY requirements as it was accepted all PfP outcomes could map to more than one TFFY outcome. For 57% of these 'maps', the PfP learning outcomes fully mapped to TFFY, 34% partially mapped and 9% slightly mapped with 23 learning outcomes (15%) not mapping at all. There were three TFFY requirements to which no PfP outcomes mapped. Of the PfP learning outcomes that fully, partially or slightly mapped, 72% were clinical skills, 16% were professional skills and 11% were blended skills. Fifty-one of the PfP outcomes mapped to a single TFFY requirement, while 98 PfP outcomes mapped to more than one TFFY requirement, as shown in Table 8.

**Table 8:** Mapping ratios of PfP learning outcomes to TFFY requirements

Mapping ratio of outcomes from PfP to TFFY	Number of TFFY outcomes
0 to 1	3
1 to 1	25
2 to 1	30
3 to 1	19
4 to 1	10
5 to 1	5
6 to 1	1
7 to 1	6
11 to 1	1
33 to 1	1

## Discussion

The publication of PfP appears to represent a significant shift in the GDC's expectations of the outcomes of undergraduate dental education. This investigation has demonstrated the changes in wording, definition and distribution, which we consider shows a change in pedagogical approach to an outcomes-based model. In PfP, there is less prescription of the systems and topics that should feature in the undergraduate curriculum for dentists. Instead, the focus is on the outcomes of dental undergraduate training which must be demonstrated to a level required for registration with the GDC - that is, the safe beginner. There is also a greater emphasis on professionalism and the skills that are overtly labelled as professionalism, communication and management and leadership - so-called 'soft skills'.^14^ The timing of publication would appear to correlate to a certain extent with an increase in published concerns regarding preparedness for practice of graduates;^5^^,^^6^^,^^7^^,^^9^^,^^10^^,^^11^^,^^12^^,^^15^^,^^18^^,^^19^ although these concerns are certainly not new.^9^^,^^16^^,^^22^ In order to understand the relationship between these two observations, it was pertinent to analyse the nature of the changes in requirements while also acknowledging the autonomy of educational providers in designing and implementing curricula which deliver intended learning outcomes.

The curriculum mapping aimed to explore changes in learning outcomes between TFFY and PfP, in addition to looking at differences in proportionality of learning outcomes defined as clinical, professional or a blend of clinical and professional skills. The mapping confirmed an overall greater number of learning outcomes for dentists in PfP than requirements in TFFY. While the number of learning outcomes describing clinical skills was similar, there were more outcomes describing professional or blended skills. This suggests there are proportionally fewer learning outcomes relating to clinical skills. In PfP, learning outcomes defining clinical skills appear to have been written to have a broader scope while those describing professional skills are more specific than those in TFFY. There were three requirements from TFFY that did not appear to have been addressed within PfP and 23 learning outcomes in PfP that may represent additional curriculum content.

The value placed on clinical experience by all stakeholders cannot be underestimated. Dental foundation trainers have cited concern over a 'reduction' in clinical experience of recent graduates compared to previous foundation trainees or their own undergraduate training; this frequently concerns more complex clinical procedures such as molar endodontics and fixed prosthodontics.^6^^,^^14^^,^^15^^,^^18^^,^^20^ Graduates also report they would have benefited from greater clinical experience,^13^ though they tend to assess their confidence levels and preparedness higher than foundation trainers.^15^^,^^19^ Ensuring the preparedness of graduates is also a priority of the GDC, though it is recognised that there are challenges to increasing the breadth and depth of clinical experience in what is considered to be 'an already full curriculum' and the extent of patient availability.^5^ In the transition to PfP from TFFY, there was an increase in the total number of learning outcomes for dentists; however, the duration of the undergraduate degree programme remained unchanged. While the number of learning outcomes referring to clinical skills has changed very little (TFFY 84; PfP 88), there are more learning outcomes describing professional and blended skills in PfP. It might be expected that with an overall increase in number of learning outcomes, dental schools have had to re-evaluate how they approach clinical skills teaching within curricula to ensure graduates can demonstrate all outcomes required by the GDC.

There is greater emphasis on professionalism in PfP learning outcomes in comparison to TFFY; this is corroborated by the increase in outcomes defined as professional and blended skills identified in the mapping undertaken by our group. While it is not clear why the GDC made such changes, it is possible that at the time of developing the PfP learning outcomes, there was an attempt to either expand professional skills deemed necessary to practise as a dentist or to provide clarification and greater detail of pre-existing expectations. Recent work on professionalism, involving a number of stakeholders, looked at the differing expectations of these groups and it is hoped the findings will be incorporated into future revisions of GDC undergraduate learning outcomes.^23^ GDC definitions of professional attributes as outcomes present additional challenges for educational providers in demonstrating attainment of complex phenomena.^24^ The shift in emphasis towards professional skills in PfP seems to be viewed negatively by those exploring graduate preparedness.^14^ However, it is the authors' opinion that the skillset of the graduate must be adaptable and reactive to the needs of the population served; professionalism and the associated skills and attributes are a core and necessary aspect of this.^25^

PfP learning outcomes describing professional skills are predominantly written with greater specificity than those requirements in TFFY. This may, therefore, account for multiple PfP learning outcomes mapping to a single TFFY requirement. In the case of the TFFY requirement 'be familiar with the legal and ethical obligations of registered dental practitioners', 33 PfP learning outcomes mapped. This suggests an expansion in detail from a broader requirement statement to a more specific and detailed learning outcome. As discussed previously, this may represent a perceived need by the GDC to be more explicit and directive in what was required, or it may be a result of the change to an outcomes-based curriculum whereby each learning outcomes needs to be assessable.

Conversely, clinical skills outcomes in TFFY were often very narrow in their scope, focusing on certain skills or procedures, thereby limiting interpretation by providers. For example, in TFFY, graduates had to 'be competent at approximal and incisal tip restorations'. This, along with other TFFY outcomes, is encompassed by the PfP learning outcome 'manage restorative procedures that preserve tooth structure, replace missing or defective tooth structure, maintain function, are aesthetic and long lasting, and promote soft and hard tissue health'. While this approach clearly provides flexibility in curriculum design for providers, there is an inevitability that individual interpretation by providers will result in graduates with a widely diverse skills and experience profile. This may also allow providers to respond to advances in technology and practice, although it is not clear if this was a motivation of the authors of PfP.

In TFFY, outcomes were preceded by a statement describing the level of understanding expected of the graduate. Graduates had to 'be competent', 'have knowledge of', or 'be familiar with' the knowledge, skill or attribute outlined in the learning outcome. This same terminology is not used in PfP, where instead there is the over-arching principle that graduates must attain the level of the 'safe beginner'. The concepts of competence, and the 'safe beginner', are open to interpretation, and are likely to vary between and within different stakeholder groups. An educational provider, for example, may aim to produce a graduate, who recognises their limitations and is comfortable to ask for help (the safe beginner), whereas a dental foundation trainer may perceive this as a graduate who lacks competence to carry out a procedure in any circumstance. In turn, this might be what has led to a perceived mismatch between what is delivered within undergraduate curricula, and what is expected of graduates in dental foundation training and beyond.^12^^,^^26^ The loss of the terminology 'be competent in', 'have knowledge of' and 'be familiar with' in PfP requires educational providers to act autonomously to decide on the level to which graduates should be able to achieve these learning outcomes. Clarification of the graduate as a safe beginner has recently become a key priority of the GDC.^5^ Alongside this, defining the purpose of undergraduate dental education would also be welcomed.^12^ The concept of a safe beginner aligns well to a model in which education and lifelong learning exist as a continuum and recognises that completion of undergraduate dental training is the starting point rather than an end point. Such a continuum requires graduates, as safe beginners, to have a view of and take ownership of their professional development, placing an importance on lifelong learning and the need for graduates to recognise and plan for their own training needs and development. All these skills are outlined much more clearly in PfP than they were in TFFY. There is an increasing body of literature in relation to preparedness, competence and confidence of undergraduates and new graduates.^6^^,^^7^^,^^8^^,^^14^^,^^15^^,^^18^^,^^19^^,^^20^ Further discussion on the use of these terms is outside the scope of this manuscript, but continued work in this area would be welcomed.

There were three requirements in TFFY to which no PfP learning outcomes mapped, as shown in Table 6. Two of these outcomes were clinical skills relating to orthodontic treatment. This almost certainly reflects a shift towards orthodontic specialists completing a large proportion of orthodontic treatment and changes to NHS contractual arrangements limiting treatment which can be provided in a non-specialist NHS primary care setting. The emphasis is now on the graduate being able to recognise when and how to refer for orthodontic assessment rather than undertake management themselves.^1^ Graduate skills in managing orthodontic emergencies and making referrals, however, are still cited in the discussion of graduate preparedness, falling below the standard expected by dental foundation trainers.^18^^,^^19^^,^^20^ The third outcome that does not feature in PfP referred to knowledge of gerodontology. With an increasingly ageing UK population, the majority of whom are increasingly likely to retain a functional dentition, it might be anticipated graduates need to have greater experience in managing this cohort of patients, or at the very least graduate with sufficient knowledge and skill in gerodontology.^27^ While PfP does make reference to the need for students to have the opportunity to practise on a wide range of patients,^1^ the lack of specific learning outcomes for treatment of the older patient may be at odds with population changes and needs.

Considering the 23 learning outcomes from PfP that could not be mapped onto requirements in TFFY, 12 of these were defined as clinical skills, therefore adding in clinically based learning outcomes. This would be counter to the argument that graduates are less well prepared but may reflect a broader curriculum, acknowledging changes in clinical practice such as technologies and disease profiles.

### Limitations

Our intention of mapping PfP learning outcomes to TFFY requirements was to provide evidence for the intended developments and change in undergraduate education; however, as previously alluded to, it is only part of the story. Mapping at this level cannot begin to explore how providers implemented PfP and the transition to an outcomes-based curriculum. Indeed, this study did not aim to understand changes to curricula by educational providers; however, it would not be unreasonable to speculate that in adding such additional content in regards to professionalism, clinical skills teaching may have had to have been reduced. At the very least, it seems probable that alternative or additional elements of assessment would have been added to most curricula to enable demonstration of attainment of the additional learning outcomes but this remains to be demonstrated.

The choice of the GDC's education documents for analysis may be a further limitation. The rationale for selection of GDC education documents for analysis was twofold. Firstly, both documents when published differed considerably from their predecessors in content and domain structure. Secondly, these documents were published in 2002 and 2011, respectively, and this timespan correlates to the period (taking into account the lag time for new intended learning outcomes to be implemented into curricula) when concerns over graduate preparedness came more frequently to the fore; the interim edition of TFFY in 2008 was not used as the learning outcomes remained the same. However, when publishing PfP, the GDC permitted a five-year grace period for providers to enact curriculum change, and it is therefore possible that for some dental schools the first students graduating from the new PfP curriculum would not have commenced dental foundation training until 2016. Therefore, we would not conclusively attribute concerns over graduate preparedness to changes in the learning outcomes. Nonetheless, it was a key event in dental education within the UK that must be borne in mind when considering these concerns.

### Implications for future iterations of GDC learning outcomes

In identifying these changes that took place in the revision of the learning outcomes from TFFY to PfP, it is evident that professionalism has become an important aspect of the undergraduate learning outcomes for dentists. It is therefore imperative that such learning outcomes are carefully considered in regard to their implementation by educational providers.^25^ We have demonstrated that there are proportionally fewer learning outcomes related to clinical skills in PfP and this continues to be a matter of concern for many of the stakeholders in dental education. It is hoped that by clarifying the definition of the safe beginner and working closely with educational providers, both undergraduate and postgraduate, the GDC will help to allay concerns over graduate preparedness. It would also be beneficial to reduce comparison of contemporary graduates to their predecessors, particularly without acknowledgement of the changing climate of dentistry. Graduates' skillsets are progressively different, but the oral health needs of the nation are also changing; graduates need skills, knowledge and experience to recognise and adapt to meet these ongoing needs in the most appropriate manner.^17^^,^^25^

## Conclusion

During the wholesale revision of requirements for undergraduate education, there has been an increase in the number of outcomes graduates must attain before they can register with the GDC. Proportionally, there are fewer learning outcomes that directly outline clinical skills in PfP than in TFFY, with the added feature of these being written with a broader scope and being subject to widely variable interpretation. Outcomes attempting to define professional attitudes and attributes are more detailed in PfP and there is a greater number of these in comparison to TFFY. This is likely to have impacted on how providers have modified curricula to meet these changes in requirements. Whether the perception of graduate preparedness can be directly attributable to these changes is not certain, but it is timely to examine these concerns to ensure graduates of the future are adequately prepared for the profession they will be working in.
